# An Efficient Spheroid Manufacturing Workflow for WJ-MSCs: Comparative Evaluation of Immediate 3D Aggregation and Conventional 2D Stabilization

**DOI:** 10.3390/bioengineering13090964

**Published:** 2026-08-23

**Authors:** Minji Ha, Joon Ho Wang

**Affiliations:** 1 Department of Health Sciences and Technology, SAIHST, Sungkyunkwan University, Seoul 06355, Republic of Korea; haminji369@naver.com; 2 Department of Medical Device Management and Research, SAIHST, Sungkyunkwan University, Seoul 06351, Republic of Korea; 3 Department of Orthopaedic Surgery, Samsung Medical Center, Sungkyunkwan University School of Medicine, Seoul 06351, Republic of Korea

**Keywords:** Wharton’s jelly-derived mesenchymal stem cell, spheroid, 3D cell culture, cryopreservation, cell therapy manufacturing, early chondrogenic priming, efficiency

## Abstract

Cryopreserved mesenchymal stem cells (MSCs) are widely used as off-the-shelf therapeutics. However, conventional manufacturing workflows typically include a post-thaw 2D monolayer recovery step before 3D spheroid formation. This study evaluated whether immediate spheroid formation following thawing (Direct-3D) could replace the conventional 2D-to-3D workflow while maintaining or improving chondrogenic potential. Using passage 5 Wharton’s jelly MSCs (WJ-MSCs), spheroid morphology, early chondrogenic priming, differentiation, and extracellular matrix (ECM) formation were systematically compared between the two strategies. The Direct-3D group exhibited faster and more uniform spheroid compaction and significantly increased *SOX9* expression as early as 3 h, indicating enhanced early priming. This response was accompanied by higher *COL2A1* expression and an increased *COL2A1/COL1A1* ratio during early differentiation while maintaining minimal collagen type X expression. These findings demonstrate that the post-thaw monolayer recovery step is not essential for producing high-quality chondrogenic spheroids from WJ-MSCs. By simplifying the manufacturing workflow while preserving—and potentially enhancing—early chondrogenic performance, the Direct-3D strategy represents a practical and scalable approach for WJ-MSC-based cartilage tissue engineering and cell therapy.

## 1. Introduction

Mesenchymal stromal/stem cells (MSCs) are multipotent stromal cells characterized by self-renewal capacity, multilineage differentiation potential, and immunomodulatory properties [1,2,3]. Because of these properties, MSCs have been widely studied as promising candidates for regenerative medicine and have demonstrated therapeutic potential in musculoskeletal, immune-mediated, and cardiovascular diseases. MSCs can be isolated from multiple tissue sources, including bone marrow, adipose tissue, and umbilical cord tissue, and each source differs in terms of cell yield, proliferative capacity, and therapeutic potential. Under appropriate induction conditions, MSCs can differentiate into osteogenic, chondrogenic, and adipogenic lineages [3,4].

Among various MSCs, Wharton’s jelly-derived mesenchymal stem cells (WJ-MSCs), isolated from the gelatinous connective tissue of human umbilical cords, are emerging as attractive cell sources for clinical applications [5,6,7]. Unlike BM-MSCs, which require an invasive and painful collection process, WJ-MSCs can be obtained from umbilical cord tissue—which is routinely discarded after birth—thereby eliminating donor-related risks and ethical concerns [6]. Compared to MSCs derived from adult tissues, WJ-MSCs exhibit higher proliferative capacity, superior differentiation potential, immunopurified properties characterized by low MHC class II expression and T cell suppression, and low immunogenicity, which are known to be advantageous for both autologous and allogeneic therapies [6]. WJ-MSCs demonstrate excellent chondrogenic differentiation potential, accompanied by increased expression of key chondrogenic markers such as *SOX9*, *COL2A1*, and *ACAN*, and are considered strong candidates for cartilage tissue engineering and regenerative applications owing to their ability to maintain low immunogenicity even after chondrogenic induction [7].

Articular cartilage is a specialized tissue devoid of blood vessels, nerves, and lymphatics, resulting in an extremely limited intrinsic capacity for self-regeneration when damaged [8,9]. Due to these characteristics, cartilage injury caused by trauma, repetitive overload, or degenerative changes tends to progress irreversibly, and along with progression to osteoarthritis (OA), constitutes a major musculoskeletal disease leading to pain, impaired joint function, and decreased quality of life, imposing a significant socioeconomic burden worldwide [9,10]. Current clinically utilized cartilage repair techniques include microfracture, autologous chondrocyte implantation (ACI), and osteochondral autograft transfer system (OATS); however, these share common limitations, such as donor site morbidity, restricted indications, incomplete tissue restoration due to fibrocartilage formation, and insufficient long-term clinical efficacy [9,11]. To overcome these limitations, cell-based cartilage regeneration strategies using MSCs are being actively investigated. Among these, WJ-MSCs, which possess both chondrogenic differentiation potential and immune-privileged properties, are emerging as a promising cell source for the development of allogeneic cartilage regeneration therapeutics [7,9].

Currently, the standard method for generating mesenchymal stem cell (MSC) spheroids typically relies on a two-step “2D-to-3D” workflow. In this standard approach, cryopreserved cells are first thawed and expanded on a 2D monolayer substrate to allow for cell recovery and stabilization; subsequently, 3D self-assembly is initiated by dissociating the cells using enzymes such as trypsin [12]. However, this intermediate step—the two-dimensional monolayer culture phase—creates a significant bottleneck in biological and translational research utilizing Wharton’s jelly-derived mesenchymal stem cells (WJ-MSCs) [13]. Following 2D stabilization, the process of forming 3D spheroids during manufacturing or mass production can lead to time delays, increased costs, and higher cell loss, and there is also a risk of cell damage due to enzymatic treatment during the thawing, reattachment, and dissociation stages. Trypsin-based enzymatic detachment has been reported to temporarily reduce or cleave cell surface adhesion molecules, including N-cadherin, which may delay initial cell–cell interactions and aggregate formation [14]. Furthermore, the abrupt transition from a rigid two-dimensional plastic surface to a three-dimensional suspension environment can induce additional mechanical and cellular stress associated with detachment, which has been reported to affect the survival and secretory behavior of MSCs [15,16]. According to previous studies, three-dimensional spheroid culture has been shown to preserve or enhance certain functional characteristics of MSCs compared to conventional monolayer culture [17].

To address these limitations, this study proposes a simplified Direct-3D suspension culture system that directly converts thawed fifth-generation WJ-MSCs into 3D spheroids without prior 2D stabilization or trypsinization. The results showed that the Direct-3D group exhibited superior initial aggregation and accelerated compaction kinetics compared to conventional methods for transitioning from 2D to 3D culture. In particular, the Direct-3D group demonstrated improved initial aggregation and enhanced early chondrogenic differentiation, as evidenced by an early increase in SOX9 expression. These results may be related to the omission of an additional enzymatic detachment step after thawing; however, cell surface adhesion proteins were not directly measured in this study. Therefore, while the Direct-3D workflow may offer a simpler manufacturing strategy (Table 1), further validation is needed to demonstrate its clinical feasibility.

## 2. Materials and Methods

### 2.1. Experimental Designs & Groups

**Figure 1:**
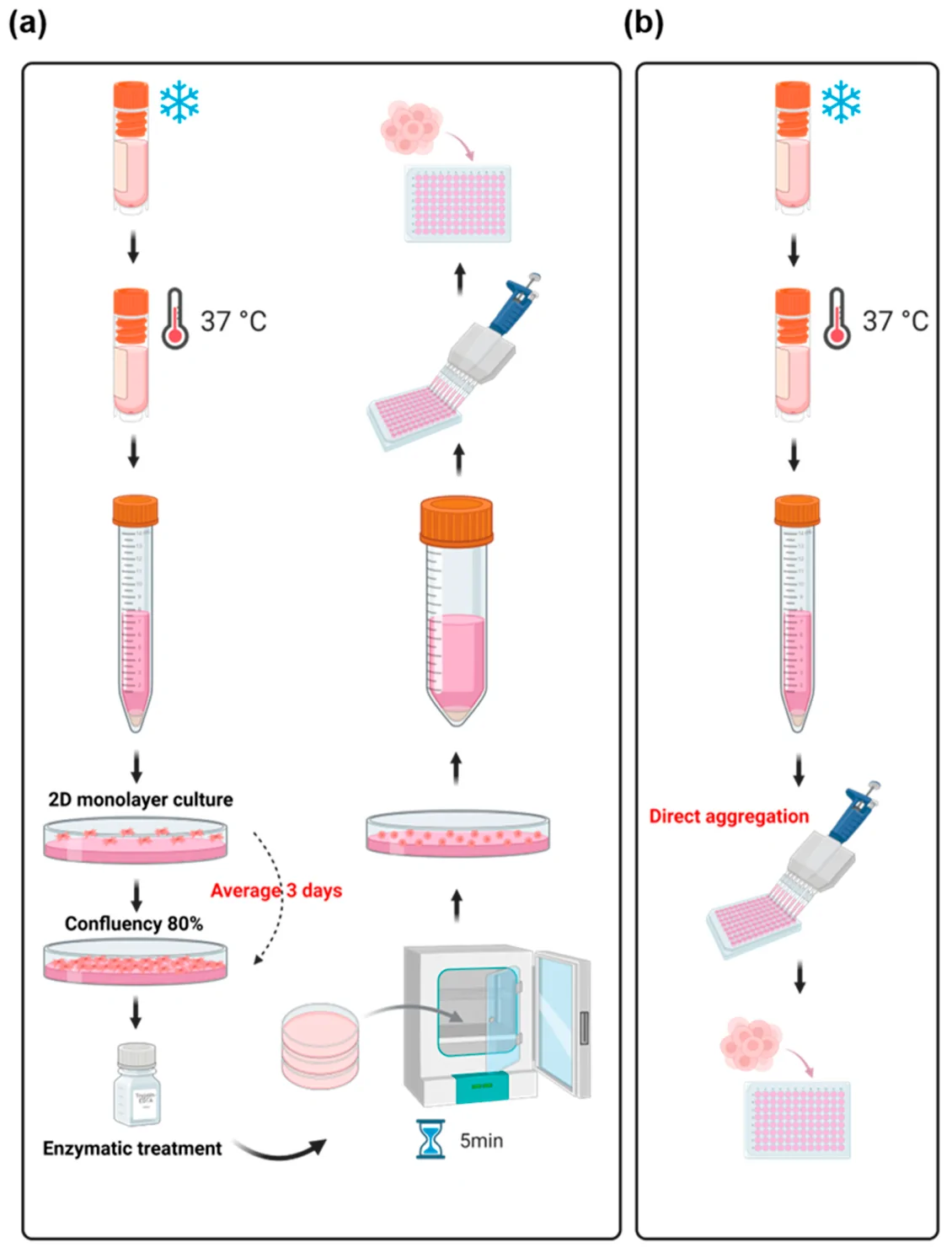
Schematic overview of the experimental design for spheroid formation from cryopreserved WJ-MSCs. (**a**) In the 2D-to-3D group, cryopreserved WJ-MSCs at passage 4 (P4) were thawed, cultured in a 2D monolayer until approximately 80% confluency, harvested by Trypsin-EDTA treatment, and then used for 3D spheroid formation at passage 5 (P5). (**b**) In the Direct-3D group, cryopreserved WJ-MSCs at P5 were thawed and directly used for 3D spheroid formation without an intermediate 2D stabilization step. After spheroid formation, both groups were cultured under identical chondrogenic induction conditions at 37 °C. Created in BioRender. K, Y. (2026) https://BioRender.com/d36sf90 (accessed on 19 August 2026).

In this study, the experimental groups were classified into two groups, Direct-3D and 2D-to-3D, according to the method used for 3D spheroid formation (Figure 1).

The 2D-to-3D group consisted of cells from the same cell stock (P4), which were thawed and seeded in a 150 mm culture dish (7.5 × 10^5^ cells/dish), cultured as a monolayer until approximately 80% confluency (2D Stabilization), collected by enzymatic treatment, and then formed into 3D spheroids at passage 5 (P5)—that is, the “post-thaw 2D stabilization followed by 3D” group.

The Direct-3D group consisted of WJ-MSC cell stock (P5) stored in liquid nitrogen (LN_2_), which was used to form 3D spheroids immediately after thawing without a 2D culture stage—that is, the “immediate 3D post-thaw” group.

To standardize passage between the two groups, the 2D-to-3D group used P5 cells obtained by thawing P4 cell stock, culturing in a 2D monolayer, and passaging through enzymatic treatment for spheroid formation, while the Direct-3D group used P5 cell stock immediately after thawing for spheroid formation. As a result, spheroids in both groups were formed from cells at the same passage (P5).

This study is a proof-of-concept (PoC) study conducted using WJ-MSCs derived from a single donor with the same genetic background to eliminate biological variation between donors (donor-to-donor variation) as an experimental variable and to precisely compare the pure effects of the cell processing procedure itself (Direct-3D vs. 2D-to-3D) on cell characteristics.

### 2.2. Cell Preparation

The cells used were Wharton’s jelly-derived mesenchymal stem cells (WJ-MSCs), and passage 5 (P5) cells were used to form 3D spheroids. Cryopreserved human Wharton’s jelly-derived mesenchymal stem cells (WJ-MSCs; Lot No. SMC-WJM-2024-003, passage 3) were provided by SMC Cell & Gene Therapy Institute, where the cells had been isolated from the Wharton’s jelly of a donated umbilical cord and expanded under Good Manufacturing Practice (GMP)-compliant conditions at the SMC Cell and Gene Therapy GMP Facility to establish a Master Cell Bank (MCB). Collection of umbilical cord tissue was performed with informed donor consent under a protocol approved by the Institutional Review Board of SMC Cell and Gene Therapy Institute (IRB No. 2016-07-102). Each cryopreserved vial contained 1.0 × 10^6^ (±20%) cells/mL. Release testing of the MCB confirmed cell viability of 86% (≥70% criterion), sterility (no growth), mycoplasma negativity, and absence of detectable adventitious virus by in vitro assay. Cell identity was verified by short tandem repeat (STR) profiling (100% match; ≥80% criterion). Immunophenotyping showed the cells were positive for CD44 (99.4%), CD73 (94.9%), CD90 (99.3%), CD105 (99.1%), and CD166 (98.1%), and negative for CD11b (0.1%), CD14 (1.9%), CD19 (0.0%), CD34 (0.0%), CD45 (0.0%), and HLA-DR (0.5%). The cells were stored frozen in liquid nitrogen (LN_2_) and rapidly thawed in a 37 °C water bath for 20–30 seconds prior to use in the experiment. The thawed cells were cultured in α-MEM (Gibco; Cat. No. 12571-048) supplemented with 10% fetal bovine serum (FBS; Gibco; Cat. No. 26140-079) and 0.5% gentamicin (Gibco; Cat. No. 15710-064). They were then centrifuged at 300× *g* for 5 min at 20 °C.

After centrifugation, the supernatant was removed, and the cell pellet was resuspended in the same medium; the cell count was determined using the trypan blue exclusion method. The cells were then seeded at a density of 7.5 × 10^5^ cells into a 150 mm culture dish and cultured at 37 °C in a 5% CO_2_ atmosphere.

### 2.3. 96well Rinsing Before 3D Spheroid Formation

Before 3D spheroid formation, a 96-well V-bottom plate was pretreated with anti-adherence rinsing solution (STEMCELL; Cat. No. 07010). We dispensed 190 µL of anti-adherence rinsing solution into each well to coat the entire plate, then removed the solution. To remove any residual solution, we washed the wells once with 230 µL of DPBS and aspirated the wash. This pretreatment was performed to create a non-adherent environment for the cells, thereby inducing uniform cell aggregation and spheroid formation.

### 2.4. 2D-to-3D Spheroid Formation

In the 2D-to-3D group, thawed WJ-MSC P4 cells were seeded in a 150 mm culture dish and cultured in monolayer for approximately 3 days to stabilize the cells. During culture, 50% of the medium was replaced the day after seeding, and when the cell density reached approximately 80% confluence, the cells were harvested by treating them with 1X (0.05%) Trypsin-EDTA (Gibco; Cat. No. 15400054) for 5 min.

The harvested cells were centrifuged, resuspended, and counted; they were then dispensed at a density of 1 × 10^5^ cells/well into a pretreated 96-well V-bottom plate. Twenty spheroids per condition were used as technical replicates, and to induce spheroid formation, the cells were centrifuged at 300x *g* for 10 min at 20 °C. The cells were then cultured at 37 °C in a 5% CO_2_ environment.

### 2.5. Direct-3D Spheroid Formation

In the Direct-3D group, WJ-MSC P5 cells stored in liquid nitrogen (LN_2_) were used for 3D spheroid formation immediately after thawing, without an additional 2D monolayer culture step. After counting the cells, the thawed cells were seeded into a 96-well V-bottom plate pre-treated to a density of 1 × 10^5^ cells/well. For each condition, 20 spheroids were used as technical replicates, and to induce spheroid formation, the cells were centrifuged at 300× *g* for 10 min at 20 °C. The cells were then cultured at 37 °C in a 5% CO_2_ atmosphere.

### 2.6. 3D Culture Conditions and Chondrogenic Induction

Immediately after 3D spheroid formation, all groups were cultured in chondrogenic induction medium (CM). A single medium change was performed the day after seeding to dilute any residual DMSO that may have remained from cryopreservation; thereafter, culturing continued with the same CM.

CM was prepared using high-glucose DMEM (Cytiva; Cat. No. SH30243.01) as the base medium, supplemented with 10 ng/mL TGF-β3 (Biotechne; Cat. No. 243-B3), 100 nM dexamethasone (Sigma; Cat. No. D4902), 50 µg/mL L-ascorbate 2-phosphate (Sigma; Cat. No. A8960), 40 µg/mL L-proline (Sigma; Cat. No. P5607), 0.5% gentamicin (Gibco; Cat. No. 15710-064), and 1% ITS supplement (Gibco; Cat. No. 41400-045). The medium was replaced every two days, and the spheroids were harvested on days 3, 7, 10, 14, and 21 after CM treatment. 

All culture processes were performed at 37 °C and 5% CO_2_, and the same culture conditions were applied to both the Direct-3D and 2D-to-3D groups. Each spheroid was maintained in an individual well throughout the culture period.

### 2.7. Morphological Analysis of Spheroids

Spheroids harvested into a 12-well plate were imaged using a 4x optical microscope, and images were acquired under identical conditions across all groups. Using ImageJ, the width and height of each spheroid were measured and defined as “*long length*” and “*short length*,” respectively. Roundness was calculated as (*short length*)/(*long length*), and the approximated diameter was defined as (*short length* + *long length*)/2. A total of 20 spheroids per group were analyzed individually.

$$Roundness=\frac{short\;length}{long\;length}$$

$$Approximated\;diameter=\frac{short\;length+long\;length}{2}$$

### 2.8. RNA Extraction and Quantitative Gene Expression Analysis

#### 2.8.1. RNA Extraction

Total RNA was extracted from single spheroids using TRIzol Reagent (Invitrogen; Cat. No. 15596026) according to the manufacturer’s instructions. The concentration and purity (260/280 ratio) of the extracted RNA were quantified using a NanoDrop spectrophotometer (Thermo Fisher Scientific, Waltham, MA, USA).

cDNA synthesis via reverse transcription was performed using the PrimeScript™ RT Reagent Kit (TaKaRa, Kusatsu, Japan; Cat. No. RR037A). A total of 100 ng of RNA was used as a template to synthesize cDNA in a 10 μL reaction mixture.

#### 2.8.2. Quantitative Gene Expression Analysis

Quantitative real-time PCR analysis using the synthesized cDNA was performed using TB Green^®^ Fast qPCR Mix (TaKaRa; Cat. No. RR430B). The QuantStudio 6 Flex Real-Time PCR System (Applied Biosystems; Thermo Fisher Scientific, Waltham, MA, USA) was used as an analytical instrument.

The genes analyzed were *GAPDH*, *COL1A1*, *COL2A1*, *COL10A1*, *ACAN*, *SOX5*, *SOX6*, and *SOX9* was used as the internal reference gene, and the relative expression levels of all genes were calculated by normalizing them to *GAPDH* expression using the $2^{-\Delta \Delta C_{T}}$ method (Table 2).

### 2.9. Alcian Blue Staining & Absorbance

#### 2.9.1. Alcian Blue Staining

Alcian blue staining was performed to confirm the accumulation of glycosaminoglycans (GAGs) within the spheroids. After treating the spheroids with 0.1N HCl, they were permeabilized at room temperature for 5 min with 10% Triton X-100 in PBS (PBS-Triton) to ensure sufficient penetration of the reagent into the spheroids, then washed with DPBS until all bubbles disappeared. The spheroids were stained in the dark with Alcian blue solution (pH 2.5, Bio Quochem, Oviedo, Spain; Cat. No. KH07001) for 1.5 h, followed by repeated washing with 0.1N HCl until the background solution became clear. The stained spheroids were imaged using a microscope.

#### 2.9.2. Absorbance

To quantify the stained GAG, the spheroids were incubated with collagenase type I (5 mg/mL) (Biochem, Lakewood, NJ, USA; Cat. No. S004197) at 37 °C for 30 min, after which 50 µL of 6 M guanidine HCl (Sigma; Cat. No. SRE0066) was added, and the spheroids were pipetted until they were completely dissolved [18].

In total, 50 µL of the dissolved sample was transferred to a 96-well flat plate, and the absorbance was measured at 650 nm using a spectrophotometer. The vehicle control, prepared with the same volume of 6 M guanidine HCl (50 µL), served as the reference.

### 2.10. Histology Analysis

HistoGel (Epredia, Kalamazoo, MI, USA; Cat. No. HG-4000-012), which had been stored refrigerated prior to harvest, was completely dissolved in a 65 °C water bath. After washing the spheroids with DPBS, 4% paraformaldehyde (Biosesang, Gyeonggi-do, Republic of Korea; Cat. No. PC2031-050-00) was added, and the samples were fixed at room temperature for 30 min before being washed with DPBS. The spheroids were embedded in approximately 200 µL of dissolved HistoGel and positioned to adhere to the bottom surface of the HistoGel. The samples embedded in HistoGel were stored at 4 °C in 70% ethanol until paraffin processing. Paraffin blocks were then prepared using standard paraffin embedding procedures. 

The paraffin blocks were sectioned at 5 µm thickness, and sections containing the spheroid center were selected for staining. Hematoxylin & Eosin (H&E), Safranin O, and Alcian blue staining were performed for histological analysis.

Quantitative analysis of sections stained with Safranin O and Alcian blue was performed using threshold-based region-of-interest (ROI) analysis with ImageJ; H&E staining was used solely for morphological evaluation. On each slide, the total area of the spheroids was defined as the ROI. To ensure analytical objectivity and strictly eliminate potential observer bias, all histological quantification was performed in a methodical, blinded fashion. Prior to analysis, all image files from both experimental groups were completely randomized and coded to mask their group identities. Specific threshold values were independently established for Alcian blue and Safranin O stainings. To determine these values without bias, a single representative image was randomly selected from the entire masked pool. The image was converted to an 8-bit grayscale format, and the ImageJ threshold range was optimized to precisely capture the stained positive area while eliminating background artifacts. Once established, this identical 8-bit threshold parameter was frozen and uniformly applied to all remaining images across all groups to calculate the positive area fraction relative to the total spheroid area. After all measurements were completed, the dataset was decoded, segregated by experimental group, and utilized for statistical analysis and graphing. Images obtained from *n* = 4 independent sections for each group and time point were analyzed, and results are presented as mean ± SEM using GraphPad Prism.

### 2.11. Immunohistochemistry Staining

Immunohistochemical staining for collagen type I, collagen type II, and collagen type X was performed on sections from paraffin blocks. The sections were deparaffinized in xylene and then rehydrated using a series of ethanol solutions (100% → 95%).

Antigen retrieval was performed using specific solutions to each target. For Collagen type I, antigen retrieval was conducted using NB600-408(Rb) (Novus Biologicals; Cat. No. NB600-408) in 0.1 M citrate (Bio Sesang; Cat. No. CR2016-050-60) at 98 °C for 10 min; for Type II collagen, NB600-408 (Rb) (Novus Biologicals; Cat. No. NB600-408) was used with 5 mg/mL hyaluronidase at 37 °C for 30 min + 0.05% pronase at 37 °C for 30 min; Type X collagen was treated with Ab49945 (Ms) (Abcam; Cat. No. Ab49945) at 5 mg/mL using hyaluronidase at 37 °C for 30 min followed by citrate at 98 °C for 10 min. After washing, the samples were incubated for 1 h at room temperature in a blocking solution consisting of 5% BSA in PBST (0.5% Tween-20) to prevent non-specific binding of antibodies.

The primary antibody was incubated overnight at 4 °C at a dilution of 1:100 in 2% BSA in PBST (0.5% Tween-20); the next day, after washing with PBS, the secondary antibody specific to the target was incubated at a dilution of 1:400 in PBS at room temperature for 30 min. The secondary antibody for Rb was Goat Anti-Rabbit IgG H&L (HRP) (Abcam; Cat. No. ab205718), and the secondary antibody for Ms was Goat Anti-Mouse Immunoglobulins/HRP (DAKO; Cat. No. P0447). Staining was performed using a DAB mixture (30 μL DAB chromogen + 1.5 mL DAB substrate). After staining, the samples were dehydrated using a series of ethanol solutions (95% → 100%) and mounted in xylene. 

Quantitative analysis of immunohistochemically stained sections was performed using threshold-based ROI (region of interest) analysis in ImageJ; ROIs were defined for COL1, COL2, and COL10 to determine the positive area ratio. On each slide, the total area of the spheroids was defined as the ROI, and a color-based threshold function was applied to isolate the positively stained regions. To ensure analytical objectivity and strictly eliminate potential observer bias, all immunohistochemical quantification was performed in an identical, blinded fashion. Prior to analysis, all image files from both experimental groups were completely randomized and coded to mask their group identities. Specific threshold values were independently established for COL1, COL2, and COL10 stainings. To isolate the true positive signals, the Color Deconvolution plugin (“H DAB” setting) in ImageJ was utilized to separate the images into distinct Hematoxylin and DAB channels. To determine these values without bias, a single representative image was randomly selected from the entire masked pool for each marker. The threshold range was optimized specifically within the isolated DAB channel to precisely capture the positive brown signals while eliminating background artifacts. Once established, this identical threshold parameter was frozen and uniformly applied to all remaining images across all groups to calculate the positive area fraction relative to the total spheroid area. After all measurements were completed, the dataset was decoded, segregated by experimental group, and utilized for statistical analysis and graphing. Images obtained from *n* = 4 independent sections were analyzed for each group and time point, and the results are presented as mean ± SEM using GraphPad Prism.

### 2.12. Statistical Analysis

Statistical analysis was performed using GraphPad Prism software. All data are expressed as mean ± SEM. Differences between groups (2D-to-3D vs. Direct-3D) and over time were analyzed using two-way ANOVA, followed by a Bonferroni post hoc multiple comparisons test. Quantitative data for each group and time point were analyzed using independent measurements from 20 spheroids for morphological analysis and 4 spheroids for all other quantitative analyses. This proof-of-concept study compared the effects of different cell processing methods on cell characteristics; all experiments were performed using WJ-MSCs derived from a single donor. *p*-values less than 0.05 were considered statistically significant.

## 3. Results

### 3.1. Morphological Characterization of WJ-MSC Spheroids

**Figure 2:**
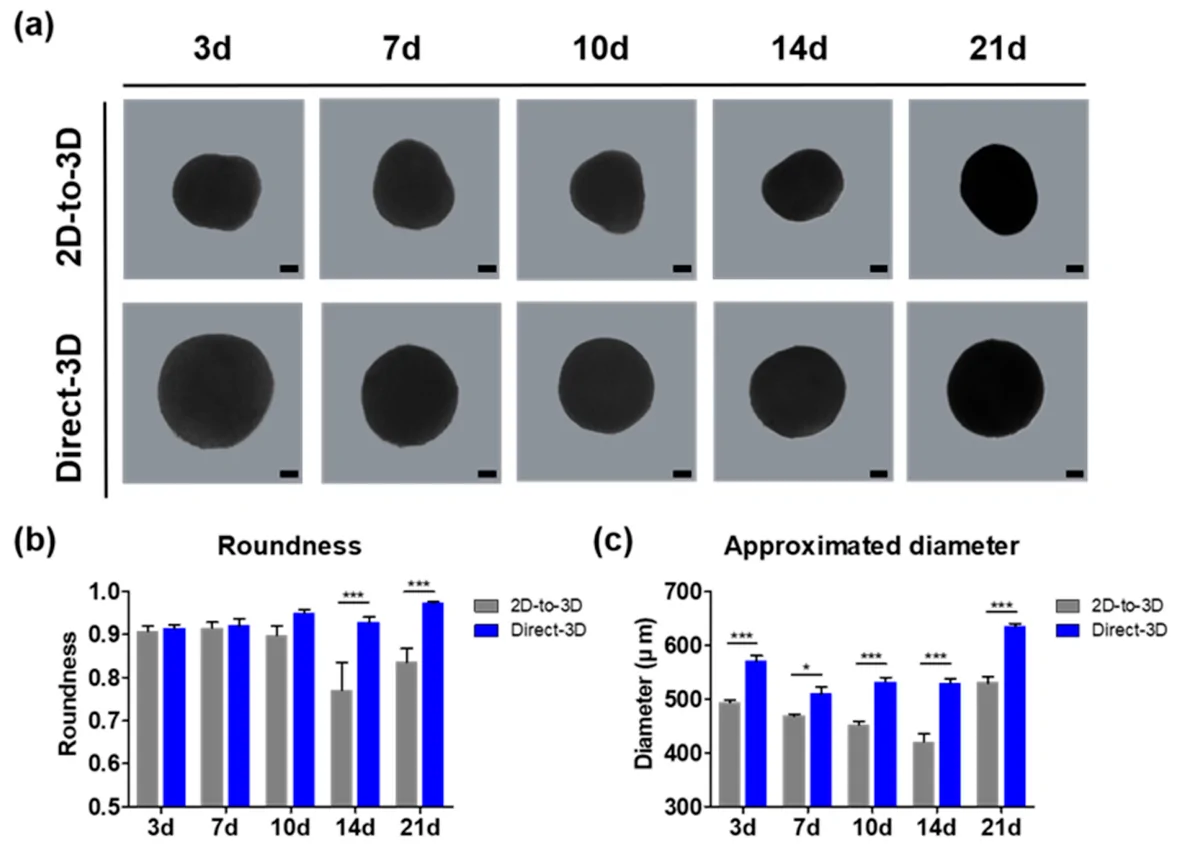
Morphological and physical characterization of WJ-MSC spheroids. (**a**) Representative images of WJ-MSC spheroids from the 2D-to-3D and Direct-3D groups at days 3, 7, 10, 14, and 21 of chondrogenic induction. Scale bar = 100 μm. (**b**) Quantitative analysis of spheroid roundness, calculated as short length/long length. (**c**) Quantitative analysis of approximated spheroid diameter calculated as (short length + long length)/2. Morphological measurements were performed using ImageJ. Data are presented as mean ± SEM. Statistical significance was determined by two-way ANOVA followed by Bonferroni’s post hoc multiple-comparisons test (* *p* < 0.05, *** *p* < 0.001).

Morphological changes in spheroids generated using 2D-to-3D and Direct-3D were evaluated throughout chondrogenic induction (Figure 2). Bright-field microscopy revealed distinct differences in spheroid morphology between the two groups. Spheroids in the 2D-to-3D group retained irregular shapes throughout the culture period and did not achieve complete compaction. In contrast, spheroids generated using the Direct-3D workflow progressively became more compact and exhibited a uniform, nearly spherical morphology over time (Figure 2a).

Quantitative analysis of spheroid roundness supported these observations (Figure 2b). The Direct-3D group showed a progressive increase in roundness throughout the culture period, approaching 1, whereas the 2D-to-3D group exhibited a gradual decrease in roundness with increased variability, particularly on day 14. These findings indicate that spheroids generated using the Direct-3D workflow maintained a more consistent morphology during chondrogenic induction.

The spheroids gradually decreased in diameter from days 10 to 14 in both groups before increasing again on day 21 (Figure 2c). Throughout the culture period, spheroids in the Direct-3D group consistently maintained larger diameters than those in the 2D-to-3D group.

### 3.2. Early Chondrogenic Priming During Spheroid Formation

**Figure 3:**
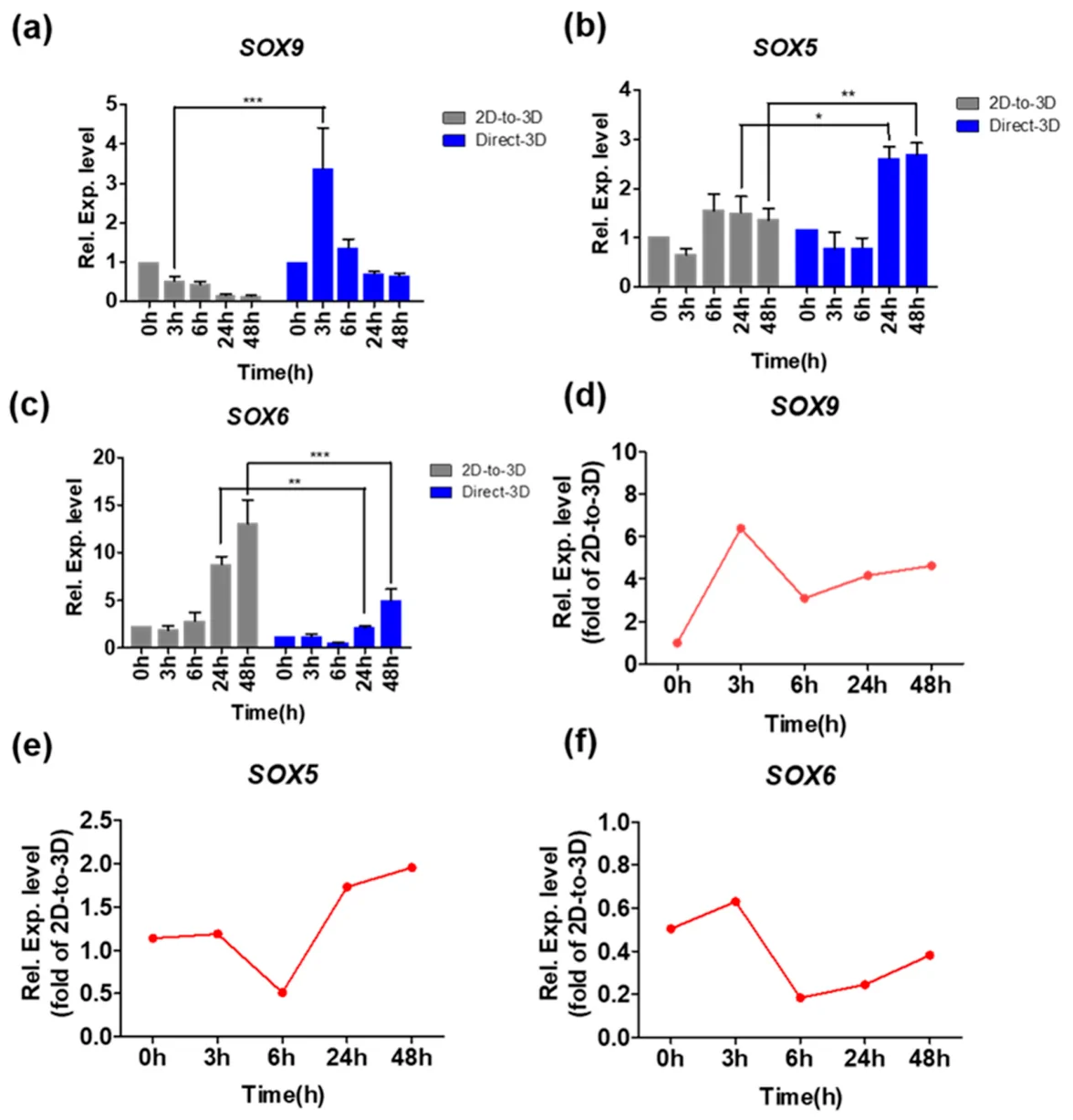
Early chondrogenic gene expression during the initial phase of spheroid formation. Relative mRNA expression levels of (**a**) *SOX9*, (**b**) *SOX5*, and (**c**) *SOX6* in WJ-MSC spheroids from the 2D-to-3D and Direct-3D groups at 0, 3, 6, 24, and 48 h after spheroid formation, as determined by qRT-PCR. Gene expression within each group was normalized to GAPDH with the mean value at 0 h set to 1, and the expression level at each subsequent time point was expressed relative to the corresponding 0 h value using the $2^{-\Delta \Delta C_{T}}$ method. Fold change of (**d**) *SOX9*, (**e**) *SOX5*, and (**f**) *SOX6* expression in the Direct-3D group relative to the 2D-to-3D group at each time point was calculated using the mean normalized value of the 2D-to-3D group as the reference. Fold-change values greater than 1 indicate higher expression in the Direct-3D group. Data are presented as mean ± SEM (*n* = 4 independent measurements per group per time point). Statistical significance was determined by two-way ANOVA followed by Bonferroni’s post hoc multiple-comparisons test (* *p* < 0.05, ** *p* < 0.01, *** *p* < 0.001).

To investigate early chondrogenic priming following spheroid formation, the temporal expression patterns of the *SOX* trio (*SOX9*, *SOX5*, and *SOX6*) were analyzed at 0, 3, 6, 24, and 48 h (Figure 3).

*SOX9* expression exhibited distinct early responses between the two manufacturing strategies (Figure 3a). In the 2D-to-3D group, *SOX9* expression gradually decreased relative to baseline throughout the early culture period. In contrast, the Direct-3D group showed a rapid increase in *SOX9* expression at 3 h, reaching approximately six- to seven-fold higher levels than the 2D-to-3D group, before gradually declining while remaining consistently higher than the 2D-to-3D group throughout the observation period (Figure 3d).

In contrast, *SOX5* expression remained relatively stable during the early culture period in both groups (Figure 3b). The 2D-to-3D group maintained comparable expression levels from 6 to 48 h, whereas the Direct-3D group exhibited a significant increase in expression between 24 and 48 h. A direct comparison between the two groups revealed similar *SOX5* expressions at 0 h and 3 h, followed by a transient reduction at 6 h in the Direct-3D group before recovery at later time points (Figure 3e).

For *SOX6,* both groups showed a gradual increase in expression over time (Figure 3c). However, the temporal patterns differed between the two groups. While the 2D-to-3D group showed a marked increase starting at 24 h, *SOX6* expression levels in the Direct-3D group remained consistently lower than those in the 2D-to-3D group at most time points (Figure 3f).

Overall, the Direct-3D workflow induced a sharp, transient increase in *SOX9* immediately after spheroid formation, while *SOX5* increased starting at 24 h and *SOX6* remained at lower levels compared to the 2D-to-3D group.

### 3.3. Longitudinal Chondrogenic Gene Expression During Differentiation

**Figure 4:**
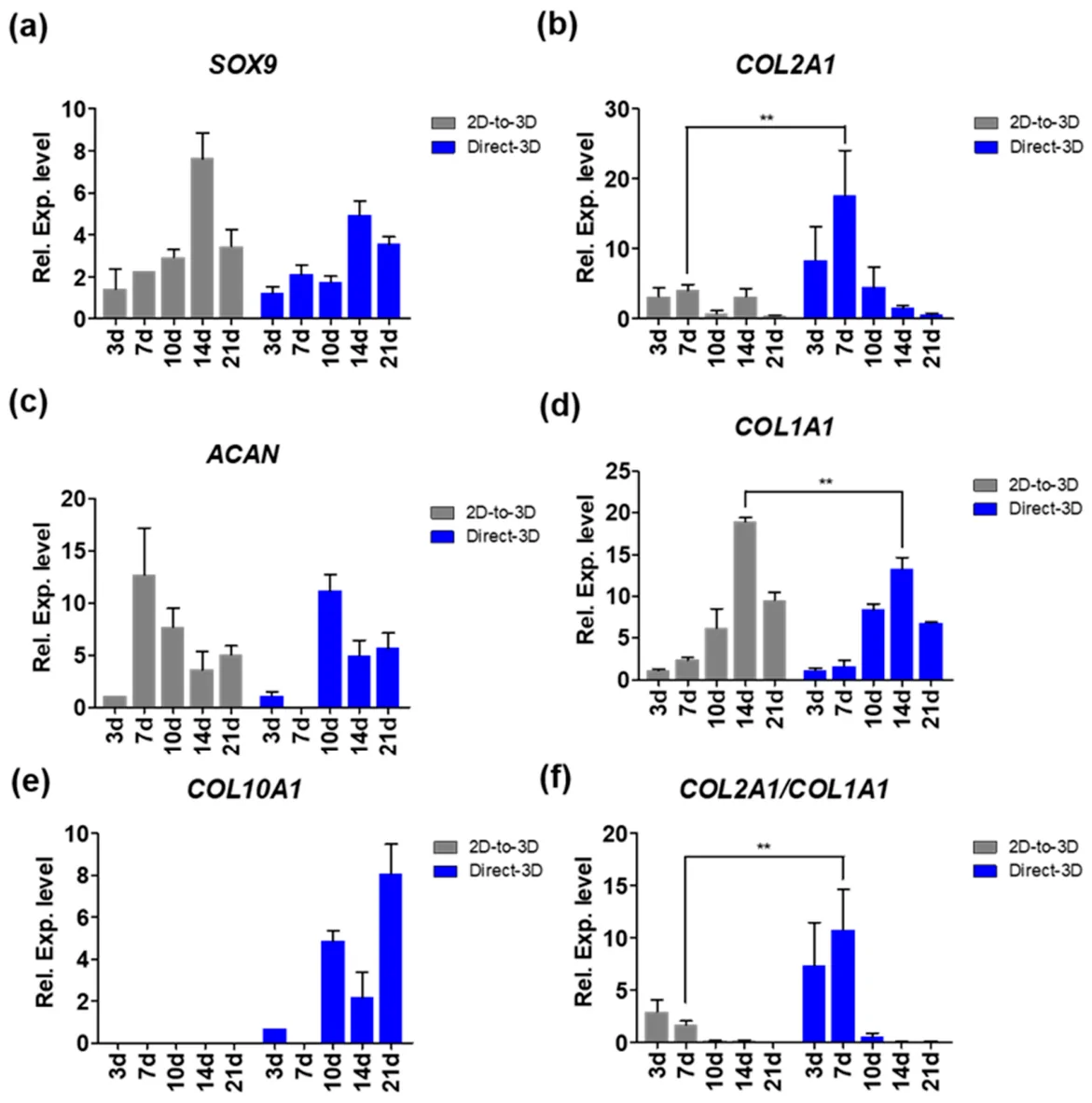
Longitudinal chondrogenic gene expression analysis during chondrogenic induction. Relative mRNA expression levels of (**a**) *SOX9*, (**b**) *COL2A1*, (**c**) *ACAN*, (**d**) *COL1A1,* and (**e**) *COL10A1* in WJ-MSC spheroids from the 2D-to-3D and Direct-3D groups at days 3, 7, 10, 14, and 21 of chondrogenic induction, as determined by qRT-PCR. Gene expression within each group was normalized to GAPDH with the mean value at 3d set to 1, and the expression level at each subsequent time point was expressed relative to the corresponding 3d value using the $2^{-\Delta \Delta C_{T}}$ method. (**f**) The *COL2A1*/*COL1A1* expression ratio, used as an index of chondrogenic matrix-related gene expression. Data are presented as mean ± SEM (*n* = 4 independent measurements per group per time point). Statistical significance was determined by two-way ANOVA followed by Bonferroni’s post hoc multiple-comparisons test (** *p* < 0.01).

To evaluate chondrogenic differentiation over time, the expressions *of SOX9*, *ACAN*, *COL2A1*, *COL1A1*, and *COL10A1* were analyzed on days 3, 7, 10, 14, and 21 following chondrogenic induction (Figure 4).

The expression profiles of the major chondrogenic markers differed between the two manufacturing strategies. *SOX9* expression followed a similar temporal pattern in both groups, remaining relatively stable from days 3 to 10, peaking at day 14, and declining by day 21. Although the overall expression levels were comparable, the 2D-to-3D group exhibited a pronounced peak at day 14 (Figure 4a).

A more distinct difference was observed for *COL2A1*, a representative marker of hyaline cartilage differentiation (Figure 4b). The Direct-3D group showed elevated *COL2A1* expression from the early stages of differentiation, with significantly higher levels than the 2D-to-3D group on day 7. In contrast, *COL2A1* expression in the 2D-to-3D group remained relatively low throughout the culture period.

*ACAN* expression remained high throughout the differentiation process in both groups (Figure 4c). The 2D-to-3D group showed a very sharp increase on day 7, while the Direct-3D group showed higher levels on day 10 than on days 3 and 7. Both groups showed a slight decline after this explosive increase.

In both groups, the levels of hypertrophic and osteogenic markers remained low. Under the current analysis conditions, *COL10A1* transcripts were not detected in the 2D-to-3D group, whereas the Direct-3D group exhibited higher expression levels and greater variability than the 2D-to-3D group (Figure 4e).

*COL1A1* expression was generally similar between the two groups, although a transient increase was observed in the 2D-to-3D group on day 14 (Figure 4d).

Consequently, the *COL2A1/COL1A1* ratio, an indicator of cartilage-specific matrix formation, was significantly higher in the Direct-3D group during the early differentiation stages (days 3 and 7) but gradually decreased thereafter (Figure 4f). In contrast, the ratio in the 2D-to-3D group remained very low and progressively decreased throughout the culture period.

### 3.4. Quantification of Glycosaminoglycan Deposition

**Figure 5:**
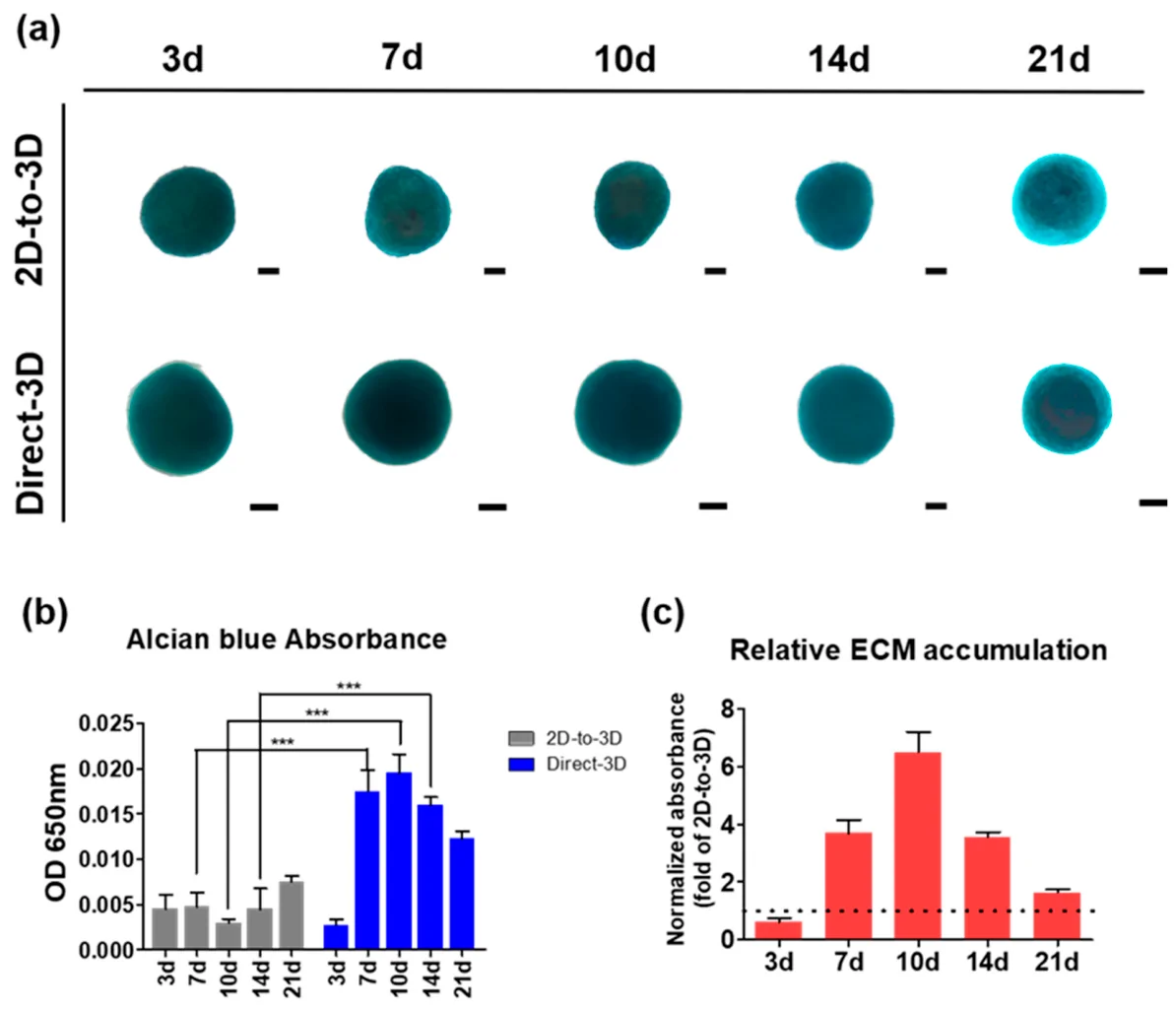
Quantitative assessment of glycosaminoglycan accumulation in WJ-MSC spheroids. (**a**) Representative whole-spheroid Alcian blue staining images from the 2D-to-3D and Direct-3D groups at days 3, 7, 10, 14, and 21 of chondrogenic induction. Scale bar = 100 μm. (**b**) Spectrophotometric quantification of Alcian blue staining at 650 nm after collagenase type I digestion and solubilization in 6 M guanidine HCl. Vehicle control (6 M guanidine HCl alone) was used as the blank. (**c**) Fold change in absorbance in the Direct-3D group relative to the 2D-to-3D group at each time point, calculated using the mean absorbance value of the 2D-to-3D group as the reference. Fold-change values greater than 1 indicate higher absorbance in the Direct-3D group. Data are presented as mean ± SEM (*n* = 4 independent measurements per group per time point). Statistical significance was determined by two-way ANOVA followed by Bonferroni’s post hoc multiple-comparisons test (*** *p* < 0.001).

To quantify extracellular matrix formation during chondrogenic differentiation, glycosaminoglycan (GAG) accumulation was evaluated using whole-spheroid Alcian blue staining and spectrophotometric analysis (Figure 5).

Representative staining results of the whole spheroids revealed distinct differences in GAG distribution between the two fabrication strategies (Figure 5a). In the 2D-to-3D group, Alcian blue staining was not uniformly distributed throughout the spheroids during culture. In contrast, the Direct-3D group exhibited a stronger and more uniform staining pattern, with the highest staining intensity observed on day 7.

Quantitative analyses confirmed these observations. The 2D-to-3D group maintained low absorbance values throughout the culture period, whereas the Direct-3D group showed a marked increase in absorbance beginning on day 7, reaching peak levels on day 10 (Figure 5b). Compared with the 2D-to-3D group, GAG accumulation in the Direct-3D group was approximately three- to four-fold higher at day 7, with the largest difference being observed at day 10 (Figure 5c).

These findings indicate that the Direct-3D workflow is associated with greater GAG accumulation during the early stages of chondrogenic differentiation.

### 3.5. Histological Evaluation of Extracellular Matrix Formation

**Figure 6:**
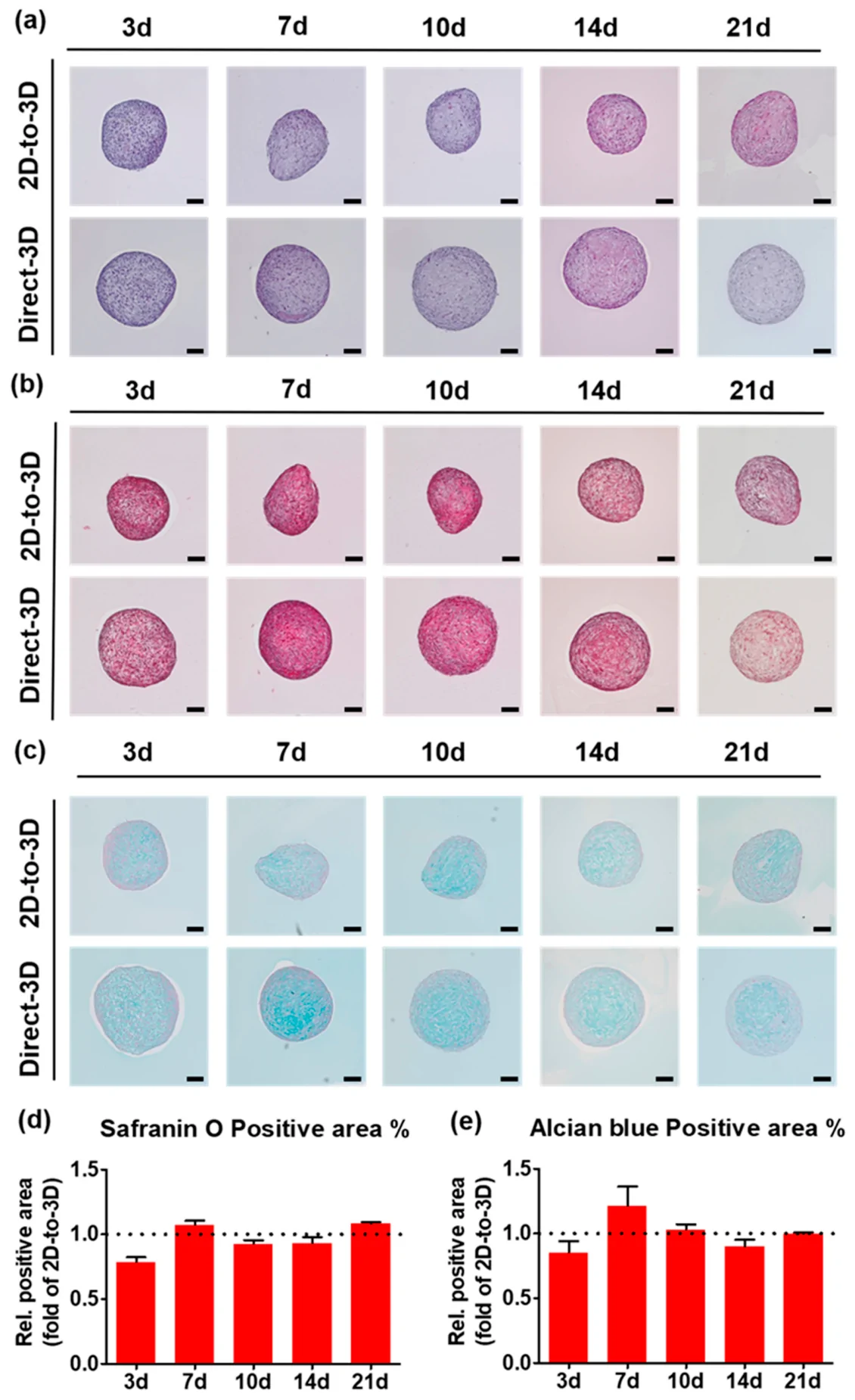
Histological analysis of extracellular matrix formation during chondrogenic differentiation. Representative (**a**) hematoxylin and eosin (H&E), (**b**) Safranin O, (**c**) Alcian blue -stained sections of WJ-MSC spheroids from the 2D-to-3D and Direct-3D groups at days 3, 7, 10, 14, and 21 of chondrogenic induction. Scale bar = 100 μm. Fold change of (**d**) Safranin O- and (**e**) Alcian blue-positive area fraction in the Direct-3D group relative to the 2D-to-3D group at each time point. Positive area fraction was defined as the ratio of positively stained area to total spheroid area and was determined using ImageJ-based color-threshold ROI analysis. Fold change was calculated using the mean positive area fraction of the 2D-to-3D group at the corresponding time point as the reference. Fold-change values greater than 1 indicate higher staining positivity in the Direct-3D group. Data are presented as mean ± SEM (*n* = 4 independent section images per group per time point).

Histological analyses were performed to evaluate extracellular matrix formation during chondrogenic differentiation using H&E, Safranin O, and Alcian blue staining (Figure 6).

H&E staining demonstrated progressive extracellular matrix deposition in both groups from day 10 onward (Figure 6a). However, distinct differences in spheroid architecture were observed between the two strategies. The 2D-to-3D group retained a heterogeneous and irregular tissue structure throughout the culture period, whereas the Direct-3D group maintained a more uniform and compact spheroid morphology from the early stages of differentiation.

Safranin O staining revealed that proteoglycans accumulated progressively in both groups during the cartilage induction period (Figure 6b). Positive staining was observed in both groups starting on day 3; however, by day 7, the staining intensity was higher in the Direct-3D group. Quantitative image analysis revealed that the safranin O-positive area was larger in the Direct-3D group at this time point (Figure 6d). Subsequently, the difference between the two groups gradually narrowed; although the 2D-to-3D group initially exhibited a larger positive area, by day 21, the Direct-3D group had a larger positive area.

Alcian blue staining results showed that glycosaminoglycan deposition gradually increased in both groups during the culture period (Figure 6c). Compared to the 2D-to-3D group, the Direct-3D group exhibited the strongest staining on day 7. Quantitative analysis revealed that, starting on day 7 and continuing throughout the remainder of the culture period (excluding day 14), the Alcian Blue-positive area was consistently larger in the Direct-3D group (Figure 6e).

### 3.6. Immunohistochemical Evaluation of Cartilage Matrix Markers

**Figure 7:**
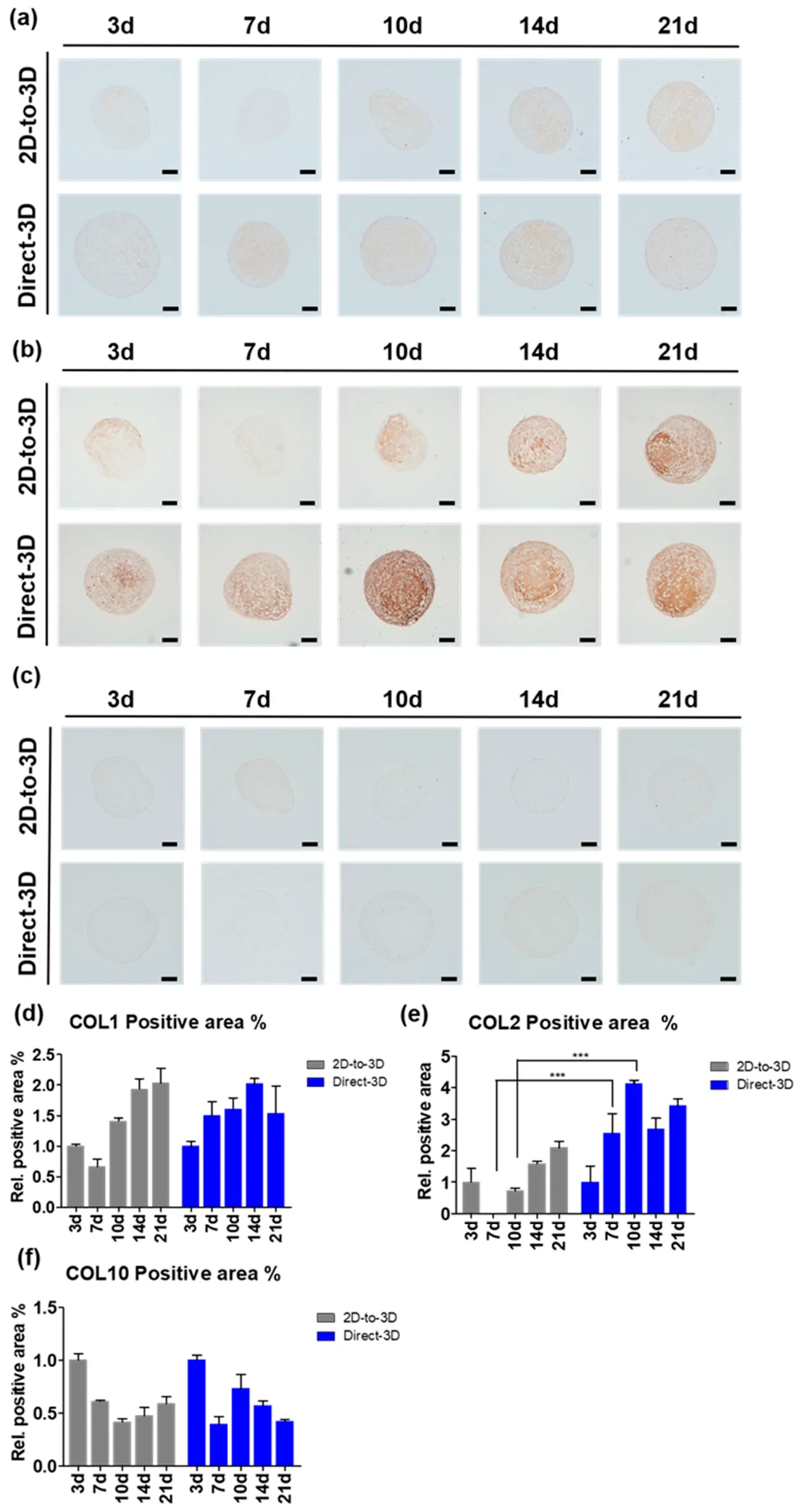
Immunohistochemistry analysis of Collagen protein expression during chondrogenic differentiation. Representative immunohistochemistry images of (**a**) collagen type I(COL1), (**b**) collagen type II(COL2), and (**c**) collagen type X(COL10) in paraffin-embedded spheroid sections from the 2D-to-3D and Direct-3D groups at days 3, 7, 10, 14, and 21 of chondrogenic induction. Positive immunoreactivity was visualized using DAB chromogen (brown signal). Scale bar = 100 μm. Relative positive area % for (**d**) COL1, (**e**) COL2, and (**f**) COL10. Within each group, the mean positive area at day 3 was normalized to 1, and the positive area at each subsequent time was expressed relative to the corresponding day 3 value. Data are presented as mean ± SEM (*n* = 4 independent section images per group per time point). Statistical significance was determined by two-way ANOVA followed by Bonferroni’s post hoc multiple-comparisons test (*** *p* < 0.001).

Immunohistochemical staining was performed to evaluate cartilage matrix formation by assessing the expression of collagen types I, II, and X during chondrogenic differentiation (Figure 7).

Collagen type I (COL1 protein) was detected in both groups throughout the entire culture period. The expression pattern showed faint signals across all spheroids in both groups, with a high percentage of positive area but no strong positive expression intensity (Figure 7a). As the culture progressed to later stages, the percentage of positive areas gradually increased (Figure 7d).

Collagen type II (COL2 protein) was detected as positive in both groups. In the 2D-to-3D group, staining intensity was low during the early stages of culture (days 3–10), but expression gradually increased as the culture progressed into the late stages (after day 14). In contrast, the Direct-3D group exhibited a higher staining intensity than the 2D-to-3D group starting from day 3. Within the Direct-3D group, staining gradually intensified starting on day 7; after reaching maximum intensity on day 10, the intensity decreased slightly before increasing again on day 21 (Figure 7b). Analysis of the positive area revealed that the 2D-to-3D group exhibited a lower positive area on day 7 than on day 3, with an upward trend starting on day 10. The Direct-3D group exhibited a higher ratio starting on day 7 than on day 3 and maintained a high level even after peaking on day 10 (Figure 7e).

In both groups, collagen type X (COL10 protein) showed minimal expression throughout the culture period (Figure 7c), and the positive area ratio was highest on day 3 in both groups; thereafter, it remained lower than that on day 3 (Figure 7f).

## 4. Discussion

Morphological analysis revealed that the 2D-to-3D group exhibited irregular shapes and high inter-spheroid variability throughout the culture period, whereas the Direct-3D group formed uniform and stable spheroid compaction, with roundness values converging to 1 as the culture period progressed. Furthermore, the increase in diameter in both groups on day 21 compared to day 14 suggests that changes in spheroid structure and potential expansion due to ECM accumulation during the late culture process cannot be ruled out [4,19]. According to previous studies, the morphological uniformity of spheroids is a key parameter for predicting quality, reproducibility, and yield during the mass production of cell therapies. Spheroids of uniform size and shape enhance the reproducibility of therapeutic efficacy by consistently maintaining intracellular nutritional and metabolic gradients [20,21,22]. In this regard, the high morphological uniformity of the Direct-3D group offers clear advantages for quality control during commercialization. 

Although both groups were compared at the same passage number (P5) for nominal 3D spheroid formation, there were design-related differences in the post-thaw processing procedures. Specifically, the 2D-to-3D group underwent additional 2D expansion and enzymatic dissociation steps, whereas the Direct-3D group was formed immediately after thawing. This raises an important question: why do such marked differences in the phenotype of 3D spheroids arise depending on the harvesting and recovery strategies? Distinct phenotypic differences, such as aggregation kinetics, structural integrity, and initial survival rates, may be attributed to several underlying biological mechanisms. First, the traditional enzymatic harvesting process using trypsin-based detachment in conventional 2D-to-3D methods may temporarily affect cell surface adhesion molecules—including N-cadherin and other surface-associated proteins essential for immediate cell–cell interactions [14]. Consequently, trypsin-treated cells may require time to recover functional cell surface adhesion, which could cause a delay in self-assembly. However, this process was not directly measured in the present study. In contrast, the Direct-3D approach omits this additional enzymatic detachment step, thereby potentially reducing damage to cell surface adhesion molecules and promoting early cell aggregation. Second, cells cultured on a rigid two-dimensional monolayer can adapt to a stiff plastic substrate through cell spreading and changes in cytoskeletal organization. Changes in the substrate’s mechanical properties, cell adhesion, and three-dimensional organization can influence MSC survival, phenotype, and secretory behavior [15,16]. Engineered three-dimensional microenvironments can regulate stem cell behavior through substrate-derived physical and biochemical signals, including conditions with limited exogenous factor supplementation [23]. Therefore, abrupt detachment of these cells from a two-dimensional substrate may impose additional mechanical and adhesion-related stress during the transition to suspension culture. However, this study did not directly evaluate anoikis-related signaling or cytoskeletal tension [15]. Since the Direct-3D workflow does not include this additional detachment step after thawing, it can reduce detachment-related stress. However, this study did not directly evaluate cytoskeletal tension, anoikis-related signaling, or the contribution of these pathways to cell survival. Finally, omitting the enzymatic detachment step may reduce the disruption of cell-associated extracellular matrix (ECM). However, since the preservation of endogenous ECM was not directly assessed in this study, this possibility remains speculative. Additional imaging or biochemical analyses would be necessary to determine whether cell-associated ECM contributes to the observed differences in aggregation [17].

In gene expression analysis, the Direct-3D group showed an approximately 6–7-fold increase in *SOX9* expression at 3 h, the early stage of spheroid formation, and maintained a gradually declining level but remained consistently higher than the 2D-to-3D group throughout the observation period. *SOX9* is a key transcription factor in chondrogenic differentiation that directly regulates the expression of cartilage-specific genes, such as *COL2A1 and ACAN* [24,25,26], and early activation of *SOX9* is an important indicator of the efficient progression of the subsequent chondrogenic differentiation program. Furthermore, *SOX5* and *SOX6* have been reported to assist *SOX9* in stabilizing the chondrogenic differentiation program [27]. Therefore, the early and sustained activation of *SOX9* observed in the Direct-3D group may have been associated with efficient chondrogenic differentiation, which is expected to have led to subsequent COL2 expression and GAG accumulation [26,28].

In contrast, while *SOX6* expression was temporarily elevated in the 2D-to-3D group at the 24 h time point, subsequent expressions of the downstream marker *COL2A1* and GAG accumulation were more pronounced in the Direct-3D group. This suggests that a transient increase in *SOX6* alone is not sufficient to lead to adequate chondrogenesis, and that the combination of *SOX9* and *SOX5/SOX6*, or the early and stable activation of *SOX9*, may be more critical determinants than *SOX6* or *SOX5* alone.

In this study, the Direct-3D group exhibited a pattern consistent with high *COL2A1* expression and the subsequent predominance of the *COL2A1/COL1A1* ratio, suggesting that the rapid initiation of early chondrogenic induction may have facilitated effective differentiation into a more hyaline cartilage-like matrix phenotype. In contrast, the 2D-to-3D group showed a gradual decrease in the *COL2A1/COL1A1* ratio as the culture period progressed. Although *SOX9* increased temporarily on day 14, *COL1A1* reached its peak level, and the *COL2A1/COL1A1* ratio reached its lowest value. This suggests that these differences may be attributable to variations in the rate of chondrogenic differentiation between groups. Indeed, previous studies have reported that decreased *SOX9* expression is associated with loss of chondral homeostasis and incomplete MSC differentiation; conversely, *SOX9* may be upregulated under conditions of unstable differentiation [28,29]. Analysis of GAG accumulation revealed that the 2D-to-3D group maintained very low levels throughout the culture period, whereas the Direct-3D group exhibited an initial surge in GAG levels, with absorbance values increasing sharply by approximately 3–4-fold by day 7, and the greatest difference was observed by day 10. This is interpreted as early *SOX9* activation, which promotes the expression of proteoglycan synthesis genes, such as *ACAN*, thereby accelerating ECM accumulation. Previous studies have also reported that *SOX9* is a key regulator of GAG synthesis, and our results support these findings [30]. This early *SOX9* activation suggests that the Direct-3D method can induce highly efficient priming of initial chondrogenesis. Therefore, given these early signals of chondrogenic differentiation, it can be confirmed that creating spheroids using early priming via the Direct-3D method—without the time-consuming 2D stabilization step— results in successful chondrogenic differentiation. This provides a far more practical, cost-effective, and time-saving strategy for cell therapy to regenerate osteoarticular tissues.

H&E staining results showed that, while the 2D-to-3D group exhibited a generally heterogeneous morphology, the Direct-3D group formed a more uniform and cohesive structure, suggesting that initial self-organization occurred more efficiently in the 3D environment [22,31]. These results are consistent with previous reports indicating that spheroid culture promotes intercellular and cell-ECM interactions that are less evident in conventional monolayer culture [32,33] and further confirm that differences also exist depending on the spheroid culture method. Safranin O staining revealed that the Direct-3D group maintained very high GAG accumulation on day 7, although the difference compared to the 2D-to-3D group tended to decrease somewhat over time. This suggests that ECM maturation and remodeling occurred during chondrogenesis after day 7 [34]. Since Safranin O is a representative staining method for evaluating chondral proteoglycan and GAG accumulation, these results support the hypothesis that more stable matrix deposition occurred in the Direct-3D group from the early stages [35,36]. Furthermore, Alcian blue staining revealed that the Direct-3D group exhibited the strongest staining intensity and the most uniform ECM distribution on day 7, suggesting that more mature cartilage matrix formation and rapid matrix remodeling occurred in the early stages [37]. Since Alcian blue is also widely used to detect acidic GAGs and proteoglycans, this supports the conclusion that early ECM accumulation was more efficient in the Direct-3D group.

Immunohistochemical analysis revealed COL1 protein expression in both groups, consistent with previous findings that in vitro chondrogenic differentiation of MSCs is accompanied by deposition of a fibrocartilage matrix [7]. In contrast, COL2 protein remained positive in both groups throughout the entire culture period; however, during the early culture phase (days 3–10), the Direct-3D group exhibited overall higher expression compared to the 2D-to-3D group, showing particularly high staining intensity on day 10. This indicates that not only was chondrogenic potential maintained, but it was also further activated during the early culture stages. In contrast, the 2D-to-3D group showed a noticeable increase in COL2 expression starting on day 14, suggesting that COL2 protein accumulation proceeded smoothly during the late culture phase (days 14–21). This suggests that, under chondrogenic induction medium conditions including TGF-β3, the *SOX9* → *COL2A1* transcriptional pathway was effectively activated in the Direct-3D group from the early stages of culture [38,39]. The co-expression of COL1 and COL2 proteins is a phenomenon commonly observed under in vitro chondrogenic conditions, suggesting that there are limitations to reproducing the complete free-floating chondrocyte phenotype in an in vitro environment [7]; at the same time, this demonstrated that even when 3D spheroids formed without a stabilization process, COL2 protein expression was sufficiently maintained and could reach high levels from the early stages. A notable finding here is that the expression of COL10 protein—an indicator of chondrocyte hypertrophy—remained at minimal levels throughout the culture period in both groups. mRNA analysis revealed high *COL10A1* expression in the Direct-3D group; however, it was confirmed that the *COL10A1* signal did not actually translate into protein expression. This suggests that there was no substantial progression toward hypertrophic differentiation. Chondrocyte hypertrophy and terminal differentiation are major concerns in MSC-based cartilage tissue engineering [40]. The fact that COL10 protein expression remained consistently low in both groups in this study suggests that the present staining results did not show substantial COL10 protein accumulation in either method under the tested in vitro conditions.

**Figure 8:**
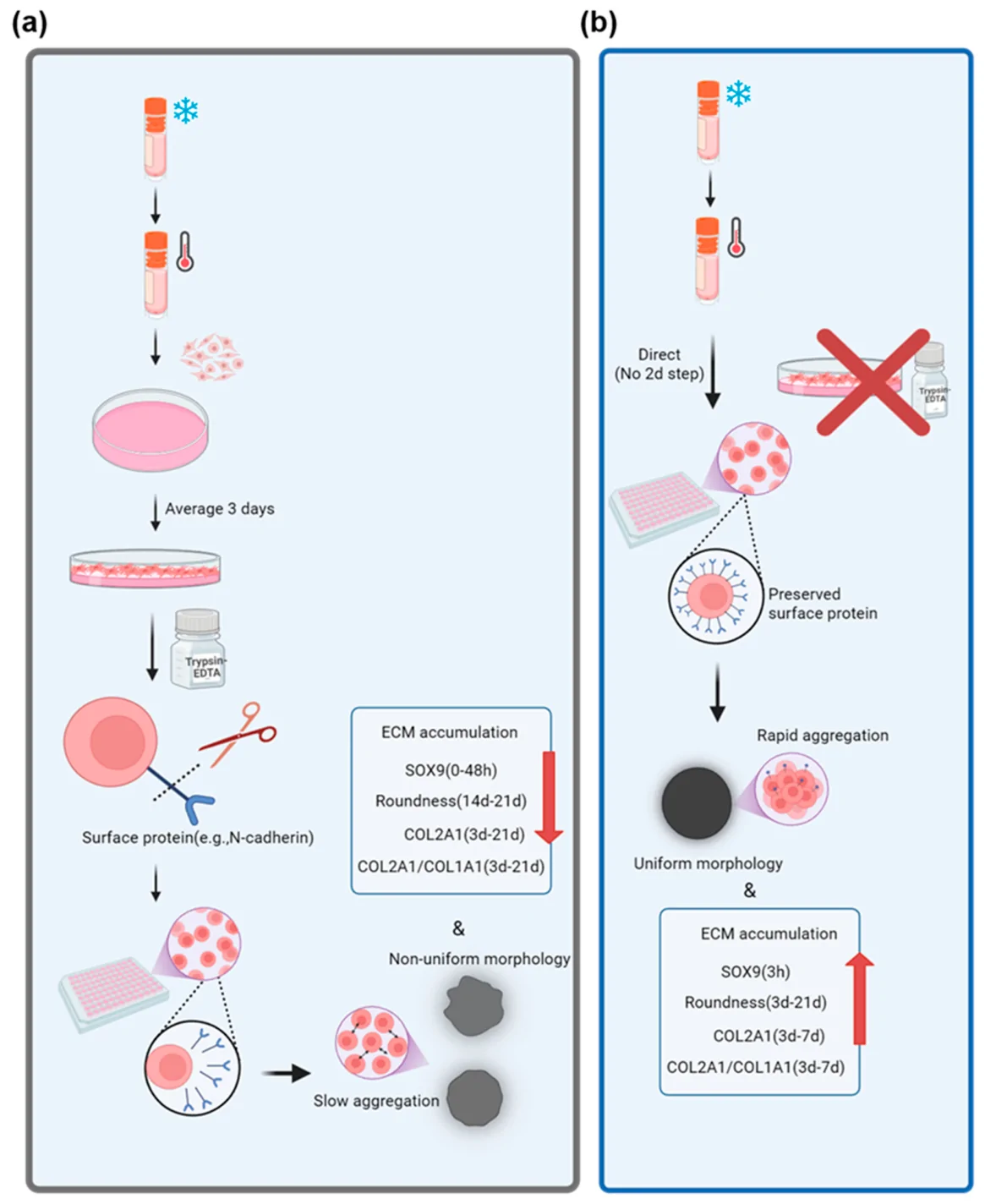
Comparative Assessment and Proposed Cellular Mechanisms of Direct-3D vs. Conventional 2D-to-3D Workflow in WJ-MSC Spheroid Manufacturing. (**a**) Conventional 2D-to-3D Workflow: Cryopreserved Wharton’s jelly-derived mesenchymal stem cells (WJ-MSCs) undergo post-thaw monolayer expansion followed by enzymatic dissociation (trypsinization). This step is hypothesized to cause transient disruption of cell-surface adhesion proteins (e.g., N-cadherin) and subject cells to potential substrate transition stress, resulting in delayed spheroid compaction, lower initial SOX9 expression, and moderate chondrogenic matrix synthesis. (**b**) Direct-3D Workflow: Thawed WJ-MSCs are immediately assembled into 3D spheroids without intermediate 2D culture or enzymatic treatment. Bypassing trypsinization is suggested to preserve functional cell-surface adhesion molecules and minimize potential detachment-associated stress. This proposed mechanism is associated with rapid cell aggregation, early SOX9 upregulation, and enhanced extracellular matrix (ECM) deposition. Created in BioRender. K, Y. (2026) https://BioRender.com/2hlez0f (accessed on 19 August 2026).

Considering these research findings, it is presumed that the differences observed between the two groups stem primarily from the differences in cell processing procedures (Figure 8). Because even relatively small differences in isolation, expansion, and banking conditions may alter WJ-MSC properties, manufacturing workflows should be standardized and evaluated under process-relevant conditions [41]. In the 2D-to-3D method, enzymatic treatment is performed to detach cells that have undergone a stabilization process after thawing from the culture dish. According to previous studies, trypsin-EDTA treatment has been reported to temporarily deplete N-cadherin, a key mediator of cell–cell adhesion, thereby delaying aggregate formation [42,43]. Furthermore, PKN2 plays a crucial role in fibroblast aggregation and spheroid formation by regulating cell motility and N-cadherin expression, supporting the view that N-cadherin-mediated cell adhesion is critical during the initial aggregation and compaction stages [43]. Trypsin’s cleavage activity is not confined to the adhesion proteins targeted during cell detachment, but extends broadly to other surface-exposed proteins involved in the regulation of cell signaling and function. As a result, enzymatic dissociation can substantially alter the overall composition of the cell surface proteome [44]. Considering these previous studies, if intercellular condensation for spheroid formation proceeds while cell surface proteins are damaged, initial aggregation may not be sufficiently activated, potentially delaying the induction of early cartilage formation. In contrast, because the Direct-3D method forms spheroids immediately after thawing, without the need for a separate two-dimensional culture or enzymatic dissociation, intercellular aggregation may occur with less exposure to enzymatic detachment; however, the extent of cell-surface protein disruption was not directly measured. Consequently, early aggregation in the Direct-3D group was likely activated more smoothly, providing a more favorable environment for subsequent compaction and early chondrogenic induction. The early and marked increase in *COL2A1* expression observed in the Direct-3D group is consistent with an environment conducive to early aggregation. However, because direct proteomic profiling or quantitative assays for cell adhesion molecules were not performed in this study, this interpretation remains speculative. We present this molecular mechanism as a strong hypothesis rather than a definitive conclusion. To validate this suggestion, further experimental verification—including Western blotting or flow cytometry to analyze N-cadherin dynamics and other functional surface receptors during the onset of aggregation—will be pursued in future research.

Previous studies have also reported that while chondrocytes rapidly undergo dedifferentiation when cultured in a 2D environment, the expression of genes related to chondrogenic differentiation increases over time in a 3D environment. Furthermore, it has been confirmed that the 3D spheroid environment itself can act as a trigger for chondrogenic differentiation, even without external differentiation-inducing factors, and that the expression of *COL2A1* and *SOX9* in a 3D culture environment is higher than in monolayer culture, regardless of the type of culture medium [19]. The results of this study are consistent with those of previous studies and are significant in that they not only confirm the role of 3D culture but also propose a new finding: the method of forming 3D spheroids itself has a decisive influence on the efficiency of the initial chondrogenic response.

From a commercialization perspective, cost reduction, time savings, and minimization of handling losses are key factors in the manufacturing and mass production of cell therapies. The 2D-to-3D method requires a multi-step process, including stabilization culture after thawing, enzymatic dissociation, and spheroid reformation. Furthermore, various losses occur due to the time required for stabilization, as well as cell stress and deterioration of cell condition during enzymatic dissociation. In contrast, the direct 3D approach eliminates these steps, simultaneously offering the benefits of process simplification, minimized handling losses, and reduced cellular stress. Crucially, these manufacturing advantages were achieved without compromising biological performance. In contrast, the direct 3D workflow demonstrated enhanced capacity to induce initial cartilage differentiation, increased extracellular matrix formation, and improved spheroid uniformity compared to the conventional 2D-to-3D approach. This combination of streamlined manufacturing and improved biological quality will promote process standardization, enhance batch-to-batch reproducibility, and support scalable, GMP-compliant production, thereby increasing the clinical application and commercial potential of MSC spheroid-based cartilage regeneration therapies. In this study, WJ-MSCs derived from a single donor were utilized. It is well-documented that WJ-MSCs can exhibit significant inter-donor variability, even under identical culture conditions and passage numbers [45]. To minimize these confounding biological variables and precisely evaluate the pure technical effects of the cell processing methods themselves (Direct-3D versus 2D-to-3D), this study was deliberately designed as a proof-of-concept investigation. Nevertheless, to generalize our findings, further validation using cells from multiple donors is essential. In future studies, we plan to confirm these results across WJ-MSCs from diverse donors, thereby demonstrating the robustness and efficiency of this streamlined process regardless of inter-donor variability.

A limitation of this study is that the exact mechanism underlying the initial surge in cartilage formation observed with the Direct-3D technique has not yet been elucidated. In particular, the molecular mechanism behind the peak in *SOX9* expression observed at the 3 h time point in the initial aggregation analysis remains a topic for future research, and further analysis is needed to confirm whether the morphological differences observed at this early point are also attributable to the same mechanism. As discussed earlier, the most likely explanation is that damage to the cell surface and junctional proteins, including N-cadherin, induced by enzymatic treatment, may have acted as a decisive factor in inhibiting early intercellular aggregation in the 2D-to-3D group [42]. Although this mechanistic interpretation is based primarily on evidence from non-MSC cell systems and was not directly measured in WJ-MSCs in this study, the observed functional results resemble the trypsin-mediated dysfunction of cell-surface adhesion proteins during the early aggregation process. Future mechanistic studies using proteomic or transcriptomic profiling at the onset of aggregation, along with real-time monitoring of intercellular clustering dynamics, would help elucidate whether the observed differences are attributable to mechanical sensing responses, the preservation of cell-surface receptor signaling, or the activation of upstream transcriptional regulators.

Furthermore, because the present study is entirely limited to an in vitro setting, comprehensive validation in in vivo implantation models remains a critical next step to fully assess its clinical applicability. Nevertheless, the observed structural and molecular differences provide a rationale for future evaluation in cartilage defect models, but their effects on graft retention, host integration, mechanical function, and tissue repair remain to be determined. Clinically, the structural properties of implanted constructs dictate their success within the harsh joint environment. The relatively compact and uniform morphology observed in the Direct-3D group may be relevant to future studies of graft handling, retention, and host integration. However, the present study did not assess mechanical properties, inflammatory resistance, graft retention, or functional cartilage repair. In addition, surface adhesion protein integrity and the composition of cell-associated ECM were not directly measured. Therefore, these possible translational advantages should be regarded as hypotheses requiring validation in appropriate animal cartilage-defect models. Our in vitro findings provide a rationale for evaluating the Direct-3D workflow in future preclinical studies.

Although the levels of cartilage differentiation in the two groups tended to converge gradually during long-term culture, the Direct-3D method exhibited a faster rate of cartilage differentiation than the 2D-to-3D method in terms of initial differentiation efficiency. This suggests that the Direct-3D method is more efficient when using spheroids in short-term culture. In other words, sufficient cartilage differentiation quality can be achieved even with a short culture period. Considering the price competitiveness and distribution efficiency of the final product, the Direct-3D method has the potential to serve as a more realistic strategy for commercialization and a practical manufacturing strategy for the cell therapy production market. In this study, we confirmed that the Direct-3D culture method exhibits faster and more robust cartilage differentiation compared to the conventional 2D-to-3D method, starting from the early stages of spheroid formation, and that cartilage differentiation proceeds effectively even without a 2D stabilization phase. This suggests that the Direct-3D method is an efficient and advantageous strategy for commercialization, offering the benefit of cost savings through reduced culture time and minimized handling processes when using early-stage spheroids.

## 5. Conclusions

This study confirmed that the “Direct-3D” method—in which spheroids are formed immediately after thawing cryopreserved WJ-MSCs—exhibits characteristics more favorable for the commercialization of early-stage spheroids in terms of initial chondrogenic potential, ECM production, and morphological uniformity compared to the “2D-to-3D” method, in which spheroids are formed after stabilization in a two-dimensional monolayer culture.

This implies that chondrogenic differentiation is possible even without 2D cell stabilization.

The Direct-3D group exhibited significantly higher *SOX9* expression than the 2D-to-3D group as early as 3 h after spheroid formation; this finding was consistent with increased *COL2A1* expression, a higher *COL2A1/COL1A1* ratio, and an early surge in GAG accumulation observed throughout the subsequent culture period.

Furthermore, the Direct-3D group maintained high morphological uniformity throughout the culture period, demonstrating characteristics favorable for ensuring quality reproducibility in the mass production of cell therapies. Although these results have not been directly verified, it is expected that when spheroids are formed without an additional enzymatic detachment step, which may facilitate early cellular aggregation, initial intercellular aggregation will be activated more efficiently, leading to successful early aggregation and rapid induction of chondrogenic differentiation.

These results suggest that the 2D stabilization step following thawing may not be an essential step in the mass production of spheroids with excellent chondrogenic potential from cryopreserved WJ-MSCs. Furthermore, they demonstrated that the Direct-3D method has the potential to serve as a more practical and efficient strategy to produce WJ-MSC spheroids in terms of process simplification, time reduction, and cost savings. By utilizing the Direct-3D method to promote early cartilage differentiation, it is expected that the total process time will be reduced, and variables related to process losses and uncertainties in estimated time arising from the 2D stabilization step will also be eliminated. However, as this study is a proof-of-concept study involving a single donor, further validation using multiple donors and in vivo models is necessary, as well as follow-up research to elucidate the molecular mechanisms of cartilage differentiation signaling pathways involved in the initial aggregation induction process. This will enable the application of these research findings to future clinical and commercial production processes.

## Figures and Tables

**Table 1:** Process characteristics and comparative analysis of 2D-to-3D and Direct-3D spheroid formation.

	2D-to-3D	Direct-3D
2D culture step	Yes	No
Enzymatic dissociation	Required	Not required
Time to spheroid	Longer	Shorter
Handling steps	Multiple	Minimal
GMP robustness	Lower	Higher

This table shows the differences in process characteristics between the 2D-to-3D group and the Direct-3D group. The 2D-to-3D group creates 3D spheroids after undergoing 2D stabilization, while the Direct-3D group creates 3D spheroids directly without a 2D stabilization process. GMP, Good Manufacturing Practice.

**Table 2:** PCR primer.

Target Gene	Sequence (5′→3′)
hGAPDH_qF	GTCTCCTCTGACTTCAACAGCG
hGAPDH_qR	ACCACCCTGTTGCTGTAGCCAA
hCOL1A1_qF	GATTCCCTGGACCTAAAGGTGC
hCOL1A1_qR	AGCCTCTCCATCTTTGCCAGCA
hCOL2A1_qF	CCTGGCAAAGATGGTGAGACAG
hCOL2A1_qR	CCTGGTTTTCCACCTTCACCTG
hCOL10A1_qF	CGCTGAACGATACCAAATGCCC
hCOL10A1_qR	TGGACCAGGAGTACCTTGCTCT
hACAN_qF	AGTAGAGGACATCAGCGGGCTT
hACAN_qR	CCGCTGATGTCCTCTACTCCAG
hSOX5_qF	CTCGGCAAATGAAGGAGCAACTC
hSOX5_qR	ACTGCCAGTTGCTGAGTCAGAC
hSOX6_qF	GCCTAAGTGACCGTTTTGGCAG
hSOX6_qR	GGCATCTTTGCTCCAGGTGACA
hSOX9_qF	AGGAAGCTCGCGGACCAGTAC
hSOX9_qR	GGTGGTCCTTCTTGTGCTGCAC

All primer sequences are written in the $5^{\prime }\;to\;3\prime$ direction. F, forward; R, reverse; h, human.

## Data Availability

The raw data supporting the conclusions of this article will be made available by the authors on request.

## Abbreviations

WJ-MSC	Wharton’s jelly-derived mesenchymal stem cell
OA	Osteoarthritis
DMEM	Dulbecco’s Modified Eagle Medium
FBS	Fetal bovine serum
RT-qPCR	Reverse transcription quantitative polymerase chain reaction
PBS	Phosphate-buffered saline
ECM	Extracellular matrix
GAG	Glycosaminoglycan
COL1	Collagen type I
COL2	Collagen type II
COL10	Collagen type X
SOX9	SRY-box transcription factor 9
SOX5	SRY-box transcription factor 5
SOX6	SRY-box transcription factor 6
ACAN	Aggrecan
IHC	Immunohistochemistry
ROI	Region of interest
ITS	Insulin–transferrin–selenium
